# Systemic therapy for recurrent and/or metastatic head and neck cancer: a population-based healthcare research study in Thuringia, Germany

**DOI:** 10.1007/s00432-021-03535-4

**Published:** 2021-01-31

**Authors:** Lisa Morkramer, Maren Geitner, Daniel Boeger, Jens Buentzel, Holger Kaftan, Andreas H. Mueller, Thomas Ernst, Orlando Guntinas-Lichius

**Affiliations:** 1 Department of Otorhinolaryngology, Jena University Hospital, Friedrich Schiller University, Am Klinikum 1, 07740 Jena, Germany; 2 Department of Otorhinolaryngology, Zentralklinikum, Suhl, Germany; 3 Department of Otorhinolaryngology, Südharz-Krankenhaus gGmbH, Nordhausen, Germany; 4 Department of Otorhinolaryngology, Helios-Klinikum, Erfurt, Germany; 5 Department of Otorhinolaryngology, SRH Wald-Klinikum, Gera, Germany; 6 University Tumor Center, Jena University Hospital, Friedrich Schiller University Jena, Jena, Germany

**Keywords:** Carcinoma, Head and neck, Recurrent disease, Metastatic disease, Palliative chemotherapy, Palliative immunotherapy

## Abstract

**Purpose:**

Systemic therapy choice for patients with recurrent and/or metastatic head and neck cancer (R/M HNC) is a challenge. Not much is known about systemic therapies used in daily clinical routine and their outcome.

**Methods:**

Data of all 283 patients with R/M HNC (89.4% male, median age: 60 years) registered for first-line systemic therapy between 2015 and 2018 in the cancer registries of Thuringia, a federal state in Germany, were included. Patient characteristics and treatment patterns were summarized. Exploratory univariate and multivariate analyses were conducted on select of systemic therapy and prognostic factors for overall survival.

**Results:**

The most frequent first-line regimens were platinum-based combinations (71.4%), mainly cetuximab + platinum + 5-fluorouracil (32.5%). 32.5, 13.1, 4.9, and 1.1%, respectively, received, a second, third, fourth, and fifth line of systemic therapy. Median follow-up was 5.5 months. Median real-world overall survival was 16.8 months [95% confidence interval (CI) 11.1–22.6]. Alcohol drinking [hazard ratio (HR) 2.375, CI 1.471–3.831; *p* < 0.001], no second-line therapy (HR 3.425, CI 2.082–5.635, *p* < 0.001), and application of three agents compared to one agent in first-line therapy (HR 2.798, CI 1.374–5.697; *p* = 0.005) were associated to decreased overall survival after start of first-line systemic therapy. Termination of second-line treatment because of deterioration of the general condition was the only independent negative prognostic factor (HR 4.202, CI 1.091–16.129; *p* = 0.037) after start of second-line systemic therapy.

**Conclusions:**

This study offers useful information, mainly prior to the availability of immunotherapy, on patient characteristics, treatment patterns, and survival in a German real-world population.

**Supplementary Information:**

The online version contains supplementary material available at 10.1007/s00432-021-03535-4.

## Introduction

More than half of patients with head and neck cancer (HNC) initially present with locoregionally advanced disease (stage III–IVb) (Chow 2020; Grunwald et al. 2020). Many of these patients develop a disease recurrence within the first 2 years following primary treatment. Primary distant metastatic HNC (Stage IVc) is uncommon (about 3.5% of newly diagnosed HNC). Patients with recurrent and/or metastatic HNC (R/M HNC) constitute a challenging population for systemic treatment because of tumor-related, patient-related and treatment-related factors (Oosting and Haddad 2019). If not amenable to curative intent treatment, the EXTREME regimen consisting of cisplatin or carboplatin with 5-fluorouracil (5-FU) and cetuximab followed by cetuximab maintenance has been the standard first-line treatment for the last decade (Vermorken et al. 2008). Head and neck cancer guidelines also recommend the inclusion of R/M HNC patients into ongoing clinical trials and, with a lower level of evidence, other chemotherapy combinations or single-agent treatment options (David et al. 2020). Recently it was shown treatment with immune checkpoint inhibitors like with anti-programmed death 1 (PD1) antibodies nivolumab or pembrolizumab improve overall survival in patients who progress after platinum containing chemotherapy compared to investigator’s choice systemic therapy (Ferris et al. 2018; Cohen et al. 2019). Recently, the Phase 3 KEYNOTE-048 trial demonstrated in the first-line R/M HNC setting, that the checkpoint inhibitor pembrolizumab in combination with chemotherapy and as monotherapy in patients with programmed death-ligand 1 (PD-L1) biomarker expression significantly improved OS compared to standard treatment with cetuximab in combination with platinum-based chemotherapy (Burtness et al. 2019). Therefore, the newest National Comprehensive Cancer Network (NCCN) guideline considers the combination of pembrolizumab + platinum + 5-FU as preferred first-line option for all patients with R/M disease who have no surgical or radiotherapeutic option (David et al. 2020). The NCCN guideline also considers pembrolizumab monotherapy as a preferred first-line option for patients with significant biomarker expression.

Actual epidemiologic population-based studies on the treatment of R/M HNC are sparse. Thuringia is a territorial state in Germany with approximately 2.2 million habitants. The Thuringian cancer registry database registers all new cases of head and neck cancer and the occurrence of R/M HNC. This provides an ideal platform for a population-based analysis of the systemic therapy in patients with R/M HNC in the years 2015–2018 in Thuringia. The focus was to collect data on current practices in daily routine and their outcome.

## Material and methods

### Ethical considerations

The Ethics Committee of the Jena University Hospital approved the study (IRB No. 3204-07/11). The Ethics Committee waived the requirement for informed consent of the patients because the study had a non-interventional retrospective design and all data were analyzed anonymously.

### Study design and patients

This population-based cohort study was based on data of the Thuringian cancer registry database. This population-based registry collects data from the five Thuringian cancer registers (Nordhausen, Gera, Suhl, Jena and Erfurt) covering all cancer cases of the federal state Thuringia, Germany, with a population of about 2 million people and covers about 98% of all head and neck cancer patients in Thuringia (Guntinas-Lichius et al. 2014). Primary head and neck cancer patients with the subsites lip, oral cavity, pharynx, larynx, nasal cavity, paranasal sinuses, salivary glands, and carcinoma of unknown primary with neck metastasis were included. Skin cancer (melanoma and non-melanoma) was excluded. This corresponded to the International Classification of Diseases, 10th version (ICD-10-GM 2020), codes C00–C14, C30–C32, C44.0–4, and C77.0. All cases with onset of a palliative systemic therapy (i.e., chemotherapy, biological therapy, and immuno-oncological therapy) treated between 2015 and 2018 (4 years) were included. Duplicate records of patients were removed. Extent of the primary disease (TNM) and of the recurrent disease (rTNM) was classified according to the AJCC Cancer Staging Classification, 7th edition (2010). Cases from 2018 originally coded based on the 8th edition were re-coded to the 7th edition to make all cases comparable. The charts of all patients including the medication plans for all lines of systemic therapy were reviewed in addition to the information of the cancer registry database. Comorbidity was calculated using the Charlson Comorbidity Index (CCI) (Charlson et al. 1994).

The epidemiological calculations were based on the average of the annual mean number of habitants in Thuringia in 2015–2018. Population numbers were used as available in the online database of the Thuringian State Office for Statistics (www.tls.thueringen.de). Incidences were calculated per 100,000 inhabitants.

### Statistical analysis

If not indicated otherwise, data are presented with mean values ± standard deviation (SD). All statistical analyses were performed using IBM SPSS, version 25. The Chi-square test was used to compare nominal data of two independent subgroups. Parameters from these univariable statistical tests with *p* < 0.05 were included into multivariable binary logistic regression models with stepwise entry to analyze independent associations and are presented with odds ratios (OR) and 95% confidence intervals (CI). Overall survival (OS) was calculated by the Kaplan–Meier method. Differences of survival were compared by the log-rank test. Multivariable analysis was performed using the Cox proportional hazards model to estimate hazard ratios (HR) with CI for OS. Nominal *p* values of two-tailed tests are reported. The level of significance for the univariate and multivariable analyses was set at *p* < 0.05.

## Results

### Subjects, prior treatment, and recurrent tumor characteristics

During the study period of 4 years, 283 patients (89.4% male, median age: 60 years) were treated. Table 1 shows the characteristics of the study cohort. The majority had no increased comorbidity (CCI = 0: 59.4%). Nearly half of the patients were smokers (43.8%). Regular alcohol drinking was also frequent (38.9%). Most of the patients received a first-line systemic therapy because of a locoregional recurrence not feasible for curative treatment (39.6%) or a progressive tumor after/under primary curative therapy (24.4%; Supplement Table 1). Oropharynx (42.8%) and hypopharynx (21.6%) were the most frequent primary tumor sites. Nearly all patients (96.1%) were treated in rUICC stage IV. The median interval between primary treatment and first-line systemic therapy was 11 months. 32% of the patients had distant metastasis (23.7% primary M +, 8.5% rM +) with pulmonary metastasis as most frequent distant metastasis location (Supplement Table 2). About one-third of the patients were chemotherapy/immunotherapy naïve, whereas the others had already received a chemotherapy as part of the primary treatment. Platinum (52.7%) and 5-fluorouracil (37.5%) were the most frequently used drugs applied in primary therapy (Supplement Table 3).

**Table 1:** Characteristics of all patients treated with systemic therapy between 2015 and 2018 (*N* = 283 patients)

Parameter	*N*	%
Gender		
Male	253	89.4
Female	30	10.6
Charlson comorbidity index (CCI)		
0	168	59.4
1	80	28.3
2	24	8.5
3	9	3.2
5	1	0.4
6	1	0.4
Age-adjusted Charlson comorbidity index (ACCI)		
0	29	10.2
1	70	24.7
2	81	28.6
3	66	23.3
4	25	8.8
5	6	2.1
6	4	1.4
8	1	0.4
10	1	0.4
Tobacco smoking		
Yes	124	43.8
No	145	51.2
Unknown	14	4.9
Alcohol drinking		
Yes	110	38.9
No	155	54.8
Unknown	18	6.4
Ethnicity		
White	283	100

	Mean ± SD	Median, range
Age, years	60.5 ± 10.0	60.0, 28–86
Charlson comorbidity index (CCI)	0.6 ± 0.9	0, 0–6
Age-adjusted Charlson comorbidity index (ACCI)	2.1 ± 2.0	2, 0–10
Smoking, pack years	4.9 ± 13.2	0, 0–60
Alcohol, drinks per day	0.7 ± 2.1	0, 0–10

*SD* standard deviation

### First-line systemic therapy

An overview of the first-line regimens is presented in Table 2. The mean incidence for initiation of a systemic therapy between 2014 and 2018 was 3.28 ± 1.31 per 100,000 persons (men: 5.92 ± 2.41; women: 0.69 ± 0.30). First-line systemic therapy was most often a combination of three drugs (42.0%) or two drugs (31.4%). The EXTREME protocol was the most frequently used regime with cisplatin (19.8%) or carboplatin (13.1%). Cetuximab monotherapy was the third leading therapy. Overall, 12 different drugs were applied. Systemic therapy was combined with palliative radiotherapy in 21.6% and with palliative surgery in 6.7% of the cases. The median duration of first-line therapy was 2.8 months. The univariate analysis showed differences between the patients receiving the EXTREME protocol and the patients receiving another regime (Supplement Table 4). CCI comorbidity was lower (*p* = 0.048), smoking and drinking was more frequent (*p* < 0.0001; *p* = 0.005, respectively), indication because of progressive disease was more frequent (*p* = 0.038), polychemotherapy as part of primary treatment as well as use of 5-FU beyond the EXTREME protocol were less frequent (*p* < 0.001; *p* < 0.001, respectively), and additional palliative radiotherapy was less frequent (*p* = 0.020) in the patients receiving the EXTREME protocol. Indication for systemic therapy because of progressive disease was the only independent factor associated with the decision for the EXTREME protocol (OR 2.665, CI 1.283–5.536, *p* = 0.009; Supplement Table 5). The reasons for the termination of the first-line systemic therapy are summarized in Supplement Table 6. Progressive disease (23.7%) was the most frequent reasons followed by termination because of deterioration of the patient’s general condition (13.4%). 19.4% of the patients still were under first-line therapy when the study was closed.

**Table 2:** First-line systemic treatment regimens

Parameter	*N*	%
Number of agents		
Single-agent regime	52	18.4
Two-agent regime	89	31.4
Three-agent regime	119	42.0
Regime unclear	23	8.1
Agent as part of the regime		
Platinum as part of regime	202	71.4
Cetuximab as part of regime	187	66.1
5-FU as part of regime	130	45.9
Taxane as part of regime	52	18.4
Nivolumab as part of regime	9	3.2
EXTREME regime		
Yes	93	32.9
No	167	59.0
Unclear	23	8.1
Regimens		
Cetuximab, 5-FU, cisplatin,	56	19.8
Cetuximab, 5-FU, carboplatin	37	13.1
Cetuximab	29	10.2
Cetuximab, carboplatin, paclitaxel	19	6.7
Cetuximab, docetaxel	17	6.0
Cetuximab, cisplatin	17	6.0
Carboplatin, 5-FU	15	5.3
Cisplatin, 5-FU	15	5.3
Cisplatin	12	4.2
Cetuximab, carboplatin	11	3.9
Nivolumab	9	3.2
Carboplatin, paclitaxel	8	2.8
Docetaxel, cisplatin, 5-FU	3	1.1
Cisplatin, etoposide	2	0.7
Carboplatin, 5-FU, paclitaxel	2	0.7
Docetaxel, cisplatin	1	0.4
Carboplatin, etoposide	1	0.4
Mitomycin C, 5-FU	1	0.4
Doxorubicin	1	0.4
Cisplatin, pemetrexed	1	0.4
Cetuximab, paclitaxel	1	0.4
Docetaxel	1	0.4
Carboplatin, paclitaxel, bevacizumab	1	0.4
Regime unclear	23	8.1
Other additional palliative treatment		
Palliative radiotherapy	61	21.6
Palliative surgery	19	6.7

	Mean ± SD	Median, range
Number of cycles in first-line therapy	3.4 ± 3.0	2, 1–21
Duration of first-line systemic therapy, months	3.7 ± 4.0	2.8, 0–24

*SD* standard deviation

### Second- to fifth-line systemic therapy

**Fig. 1:**
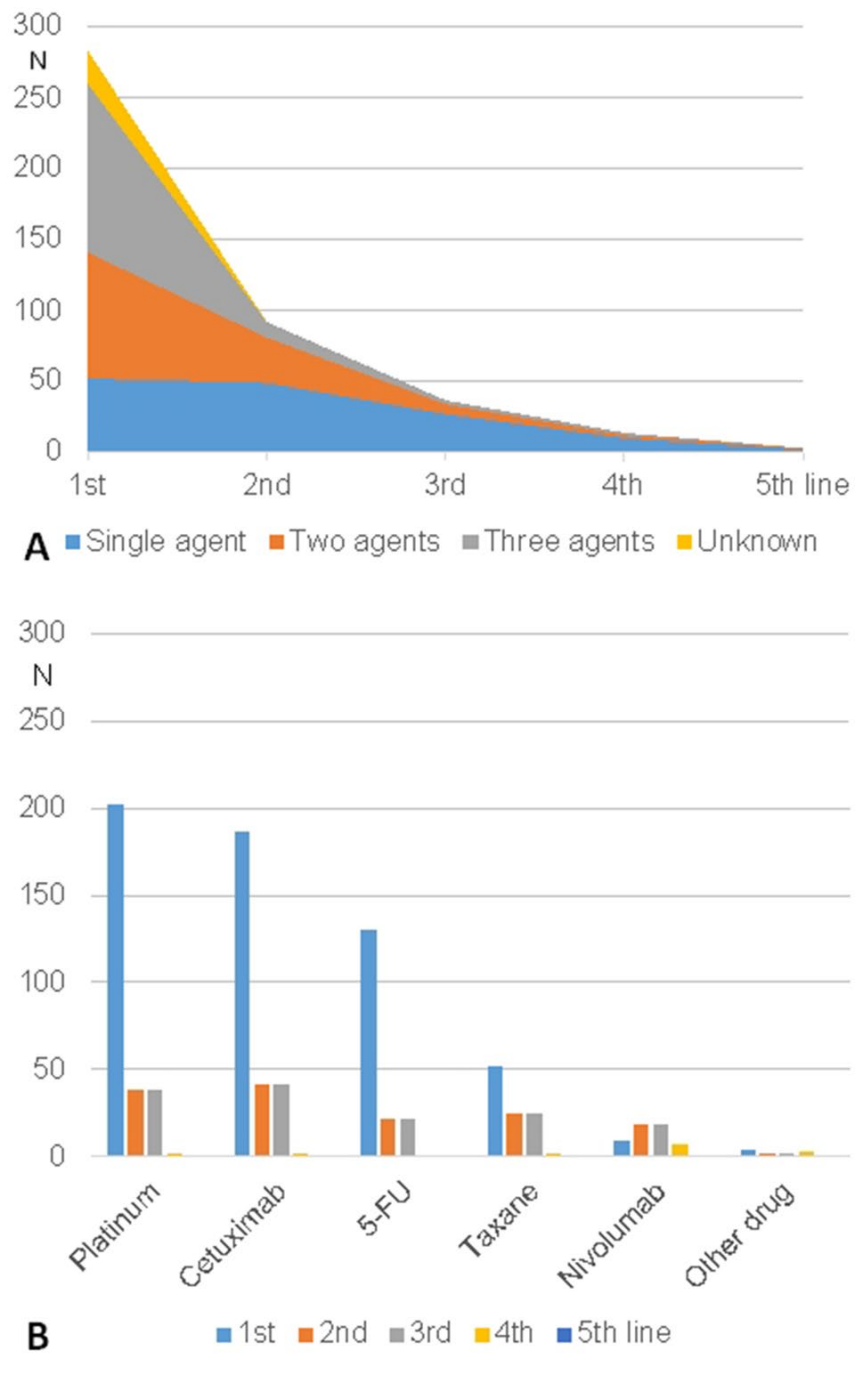
Systemic therapy patterns in absolute numbers. **a** Use of singe versus combined agent regimes over the sequence of systemic therapy lines. **b** Use of the most important agents over sequence of systemic therapy lines

32.5, 13.1, 4.9, and 1.1%, respectively, received a second, third, fourth, and fifth line of systemic therapy. The use of a combination of three or two agents decreased for second and further line (Fig. 1a). Platinum, cetuximab, and 5-FU were the dominating agents in first- and second-line therapy (Fig. 1b). The use of taxanes and nivolumab increased with second and third-line. 32.5% of the patients received a second-line systemic therapy, and 13.1% even a third-line therapy. More data on the second- to fifth-line systemic therapy are given in Table 3. Single drug regimens were dominating. Data on lines, cycles and agents for all up to five lines of systemic therapy are shown in Supplement Table 7. When comparing only the patients with completed first-line therapy, some differences between the patients receiving a second-line therapy compared to the patients not receiving a second-line therapy become obvious (Supplement Table 8). Smokers (*p* = 0.039), patients with tumor of the oral cavity (*p* = 0.042), patients with termination of first-line not because of deterioration of the general health condition (*p* = 0.001), patients with first-line with three agent regime (*p* = 0.022), and platinum as part of the first-line regime (*p* = 0.013) received more frequently also a second-line systemic therapy. Termination of first-line not because of deterioration of the general health condition (OR 1.912, CI 0.948–3.854; *p* = 0.001) and platinum as part of the first-line regime (OR 2.863, CI 1.009–8.126; *p* = 0.048) remained independent factors associated with decision for second-line therapy in the multivariate analysis (Supplement Table 9).

**Table 3:** Second-line to fifth-line systemic treatment regimens

Parameter	2nd line		3rd line		4th line		5th line	
	*N*	%	*N*	%	*N*	%	*N*	%
Further lines of systemic treatment								
Yes	92	32.5	37	13.1	14	4.9	3	1.1
No	191	67.5	246	86.9	269	95.1	280	98.9
Number of agents								
Single-agent regime	49	17.3	27	9.5	10	3.5	2	0.7
Two-agent regime	32	11.3	7	2.5	2	0.7	1	0.4
Three-agent regime	11	3.9	3	1.1	2	0.7	0	0
Agent as part of the regime								
Platinum as part of regime	38	13.4	38	3.5	2	0.7	1	0.4
Cetuximab as part of regime	41	14.5	41	2.8	2	0.7	0	0
5-FU as part of regime	22	7.8	22	1.1	1	0.4	0	0
Taxane as part of regime	25	8.8	25	2.1	2	0.7	1	0.4
Nivolumab as part of regime	19	6.7	19	3.9	7	2.5	1	0.4
Other* agents	2	0.7	2	2.5	3	1.1	1	0.4
Other additional palliative treatment								
Palliative radiotherapy	14	4.9	3	1.1	3	1.1	0	0
Palliative surgery	5	1.8	2	0.7	1	0.4	0	0

	Mean ± SD	Median, range	Mean ± SD	Median, range	Mean ± SD	Median, range	Mean ± SD	Median, range
Number of cycles	5.0 ± 4.5	4, 1–24	4.1 ± 3.0	3.5, 1–12	7.8 ± 7.4	6, 1–20	3 ± 0	3, 3
Duration systemic therapy, months	3.7 ± 4.5	3, 0–29	3.1 ± 2.6	4, 0–9	4.1 ± 2.5	3, 2–8	2.0 ± 2.0	2, 0–4
Interval primary treatment to line of systemic therapy, months	23.9 ± 27.4	14, 171	28.7 ± 25.3	19.5, 3–128	33.5 ± 23.5	27, 15–95	33.3 ± 5.5	31, 26–37

*SD* standard deviation; other agents: 2nd line: methotrexate, doxorubicin; 3rd line: Temsirolimus, pembrolizumab, vinorelbine, methotrexate; 4th line: Epirubicin, cyclophosphamide, vincristine, mitomycin C, temsirolimus; 5th line: Gemcitabine

### Overall survival under palliative systemic therapy

**Fig. 2:**
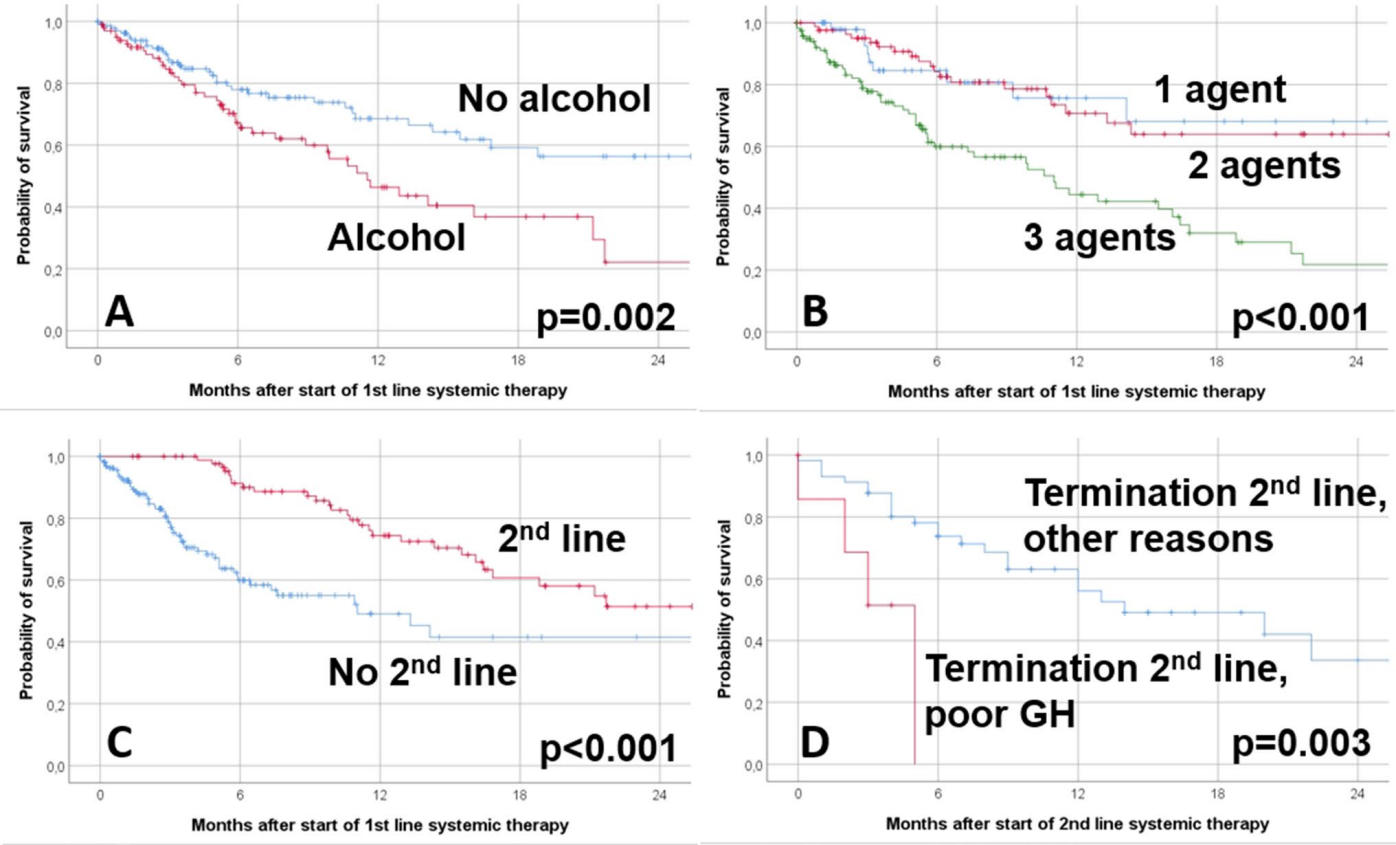
Kaplan–Meier curves of overall survival and log-rank test results for different prognostic factors during first-line therapy (**a**–**c**) and during second-line therapy (**d**): **a** Alcohol drinking. **b** Singe or combined used of agents for systemic therapy. **c** Second-line therapy. **d** Reason of termination of the second-line therapy GH = general health

At the end of the study period, 67.8% of the patients were alive (Supplement Table 10). The median interval between diagnosis of the initial primary HNC and diagnosis of the R/M HNC was 18.2 months. Median follow-up since start of first-line systemic therapy was 5.5 months. Median overall survival since initial diagnosis of the primary tumor was 66.7 months (CI 42.5–90.9). Median overall survival since start of first-line systemic therapy was 16.8 months (CI 11.1–22.6). The 6-month and 12-month overall survival rates after start of first-line systemic therapy were 73.3% and 59.6%, respectively. The probability of survival after start of first and further lines is shown in Supplement Fig. 1. The probability of survival after initial diagnosis of the primary tumor and some prognostic factors (other data not shown in relation to start after primary treatment) is presented in Supplement Fig. 2. The univariate analysis of prognostic factors for overall survival after start of first-line systemic therapy is shown in Supplement Table 11. Impaired overall survival was associated with several factors such as: alcohol drinking (*p* = 0.002), no polychemotherapy as part of treatment of the initial primary HNC (*p* = 0.013), no treatment with 5-FU as part of initial primary treatment (*p* = 0.008), a three agent regime for first-line systemic therapy (*p* < 0.0001), platinum in first-line (*p* = 0.015), cetuximab in first-line (*p* = 0.047), 5-FU in first-line (*p* < 0.0001), ≤ 2 cycles of first-line (*p* = 0.005), duration of first-line < 2.8 months (*p* = 0.039) other reason than progressive disease for termination of first-line (*p* = 0.006), and no second-line (*p* < 0.0001) (Fig. 2a–c). Two different multivariate models are presented in Supplement Table 12 and Supplement Table 13. The first model looked at the relevant parameters of the first-line therapy in more detail. Here, alcohol drinking (HR 3.165, CI 01.420–7.042; *p* = 0.005) and no second-line therapy (HR 11.493, CI 1.150–114.89; *p* = 0.038) were independently associated with worse overall survival. In the second model, alcohol drinking (HR 2.375, CI 1.471–3.831; *p* < 0.001) and no second-line therapy (HR 3.425, CI 2.082–5.635, *p* < 0.001) remained independent negative prognostic factors. In addition, the application of three agents compared to one agent as part of first-line systemic therapy was associated to decreased overall survival (HR 2.798, CI 1.374–5.697; *p* = 0.005).

Median overall survival after start of second-line therapy of the subgroup of patient receiving a second-line therapy was 20.0 months (CI 10.2–29.8). The 6-month and 12-month overall survival rates after start of second-line therapy were 73.3% and 59.6%, respectively. When focusing the univariate analysis on the second-line therapy, smoking (*p* = 0.040), drinking (*p* = 0.004), three agents as part of first-line systemic therapy (*p* = 0.007), the EXTREME protocol as first-line systemic therapy (*p* = 0.028), a second-line with three agents (*p* = 0.018), and termination of second-line because of deterioration of the general condition (*p* = 0.003) were associated to decreased overall survival (Fig. 2d; Supplement Table 14). In the multivariate analysis (Supplement Table 15), termination of second-line treatment because of deterioration of the general condition remained the only independent negative prognostic factor (HR 4.202, CI 1.091–16.129; *p* = 0.037).

## Discussion

Although palliative systemic therapy forms unfortunately a substantial part of the treatment of patients with R/M head and neck cancer, no international uniform treatment guideline exists. The results of the study showed a wide variation of regimens and drugs used for systemic therapy. Platinum-based combinations were most frequently used as first-line therapy (71.4%). Due to a recent survey on palliative treatment for head and neck cancer, the EXTREME protocol was the institutional standard of care for first-line treatment in most centers in Germany in the period of the present study (Laban et al. 2016). Nevertheless, the EXTREME protocol was used only for 32.9% of the patients for first-line systemic therapy. Taking into account the low comorbidity rate, this was surprising. Due to the retrospective design of the study, the reasons why not more patients received an EXTREME treatment that was considered standard in the study period, remain unclear. Furthermore, the recommendation from clinical trials is to give six cycles followed by maintenance of cetuximab (Vermorken et al. 2008). In this study presenting real-word data, only 8.6% of the patients with EXTREME first-line treatment received six cycles. Beyond progressive disease, the treatment was terminated in about 30% of the cases because of intolerance, allergy, side effect, or deterioration of the patient’s general condition. This cannot explain the early termination for all cases, but shows that the real-life settings do not guarantee an optimal setting to get most patients through the recommended number of cycles.

32.5% of the patients received a second-line therapy and even 13.1% a third-line therapy. Single-agent regimens were the most frequent second-line therapy (53% of all second-line therapies). Cetuximab, platinum, and taxanes were the three most used single agents for second-line therapy (45, 41, 27%, respectively, of all second-line therapies). Nivolumab was licensed in 2017 and pembrolizumab in 2019 for treatment of R/M head and neck cancer. This explains the low number of second-line treatments with nivolumab (21% of all second-line therapies), because treatment with this checkpoint inhibitor only started in 2017. Overall, the drugs and combinations used for first and further lines of systemic therapies for patients with R/M HNC varied significantly across the analyzed population. Such a high variation of different regimens was recently also across some countries in Europe, Asia Pacific and Latin/North America (Grunwald et al. 2020). A web-based survey performed between 2013 and 2014 on palliative treatment for head and neck cancer in German-speaking countries also revealed a large variation but no standards especially for second- and third-line treatments. The authors assume that reasons for this could be the physicians’ individual experience as well as the varying assessment regarding the toxicity of palliative systemic therapy (Laban et al. 2016). The valid NCCN guidelines for the years 2015–2018 clearly favored a combination of platinum, 5-FU, and cetuximab ahead of other combinations or a single-agent option (Adelstein et al. 2017). Nevertheless, the NCCN guideline and German guidelines do not give clear criteria for or against a specific protocol (Wolff et al. 2012; Bootz 2020). Not very old patients or patients with (more often felt than measured) better performance status might receive more aggressive protocols and more frequently combination therapy (La et al. 2018). This large scope for decision making might be the reason for the high variation of different systemic therapy regimens.

Median overall survival after start of first-line systemic therapy was 16.8 months. This is much longer than other recently published German real-world data from 2011 to 2013 in sample with comparable treatment regimens: 102 patients with probably lower general health status (ECOG performance status ≥ 2: 41%) than in the present study showed a median overall survival of only 7 months (Grunwald et al. 2020). In contrast, a retrospective data collection of 462 patients from a network of community oncology practices in the United States from 2007 to 2015 on effectiveness of systemic therapy for R/M HNC estimated a median overall survival of 21.0 months, i.e., even longer than in the present study (Fisher et al. 2018). In contrast, in trials on first-line systemic therapy, typically patients with an ECOG performance status > 1 are not included. In these studies, median overall survival ranges from about 8.2–10.7 months for cetuximab with chemotherapy, reach about 14.9 months for checkpoint inhibitors, and reach maximally 6.9 months for other single-agent regimens (Vermorken et al. 2008; Burtness et al. 2019). A direct comparison of the data is not possible. We can only conclude that first-line systemic therapy with the used protocols seemed to be, in general, effective in the clinical routine setting. The same holds true for second-line treatment. Median overall survival after start of second-line systemic therapy was 20.0 months. In trials using checkpoint inhibitors, median overall survival was about 7.5 months, and from 5.1 to 6.9 months for other combinations or monotherapies (Ferris et al. 2016; Pai et al. 2019; El Rassy et al. 2019). It is an important limitation of the present study that we cannot verify the correctness of the indication for any line of systemic therapy in retrospect. The series might include outliers with good prognosis not requiring systemic therapy.

A small amount of patients (12.4%) of the present study received the systemic therapy as primary treatment for stage IVc cancer. Overall survival of these patients was not significantly different from the patients receiving systemic therapy for a recurrent disease. In a recent population-based study analyzing patients from the National Cancer Database treated between 2003 and 2006 for primary stage IVc head and neck cancer, a 6-month and 12-month overall survival rate of about 70% and 50% was estimated, i.e. equivalent to the present data. Unfortunately, the drugs used for systemic therapy were not reported (Schwam et al. 2015).

Although presenting a large series of real-word data, the present study has important limitations. Due to the retrospective design, decision making for or against the selected systemic therapy regime remained unclear. The incidence of systemic therapy for HNC was 3.28/100,000 persons. The average incidence of newly diagnosed HNC in the years before was about 16–17/100,000 persons in Thuringia (Dittberner et al. 2020). Hence, about 20% of the patients receive directly or later on a systemic therapy for R/M HNC. If this proportion is low or high, cannot be answer as comparable study are missing. Standardized data on the performance status of the patients were missing. This might be the reason why the calculated comorbidity was low in our study sample (59.4%). It might be that the comorbidity was underestimated due to missing data. Alternatively, the study sample might represent a positive selection of good performers selected for systemic therapy, whereas bad performers might have had a lower probability to receive a systemic therapy. Instead, such patients might have had a higher probability to be selected for best supportive care and were not covered by the present study. Furthermore, the HPV status of the patients was unknown. It should, however, be stated that the HPV status does not play a role for treatment selection in the R/M HNC setting (Misiukiewicz et al. 2014). Furthermore, it seems that the HPV status has no predictive role when choosing a checkpoint inhibitor for treatment of R/M HNC. The prognostic role of HPV when using immunotherapy in the R/M HNC setting is undetermined (Bauml et al. 2017; Ferris et al. 2018; Cohen et al. 2019). An important strength of the study was the combination of population-based data and hospital-based data. The population-based approach allowed a representative reflection of the health care of R/M HNC patients in daily routine beyond clinical trials. The addition of data of the charts of all patients allowed a detailed analysis of the regimens, lines and cycles. This would not have been possible based on clinical cancer registry data.

The present study ended when a new era of systemic therapy just started. The phase 3 KEYNOTE-048 trial showed that pembrolizumab in combination with chemotherapy or as monotherapy improved overall survival compared to platinum-based chemotherapy with cetuximab in the first-line R/M HNC setting (Burtness et al. 2019). Therefore, pembrolizumab alone or in combination with chemotherapy is considered as the new first-line standard by many head and neck surgeons and oncologists. Cetuximab in combination with platinum-based chemotherapy is seen important in the future only for patients with no PD-L1 expression and as second-line treatment and all single-agent regimes for second or even third-line therapy. Fit patients with no PD-L1 expression might be good candidates for the TPExtreme protocol combining cetuximab with platinum and docetaxel (instead of 5-FU) (Guigay et al. 2019). We should not forget that only a minority of R/M HNC patients responds to immune-oncology therapies. Some important phase III trials are ongoing, for instance, the CheckMate 651 trial of nivolumab in combination with ipilimumab (NIH U.S. National Library of Medicine 2020), and the KESTREL trial of another PD-L1-inhibitor, durvalumab monotherapy or durvalumab in combination with tremelimumab (NIH U.S. National Library of Medicine 2020). The task now will be to collect and analyze large real-word dataset of patients treated with immune-oncology therapies.

## Conclusion

This study provides actual population-based information on characteristics of patients with R/M HNC, systemic therapy treatment strategies, and overall survival in a real-world setting of a federal state in Germany from 2015 to 2018, i.e., at the transition of the pre-immunotherapy to the immunotherapy era. The patterns of first-line, second-line and further lines of systemic therapy varied among the patients reflecting the urgent need for clinical standards, for instance by national or international clinical guidelines. Overall survival in the palliative setting under systemic therapy improved in the recent years. Future real-world population-based research should analyze if the introduction of immune-oncology therapies provide meaningful improvement of outcome for patients with R/M HNC.

## Supplementary Information

Below is the link to the electronic supplementary material.

Supplementary file1 (DOCX 77 KB)

Fig. 1. Kaplan-Meier curves of overall survival after start of different lines of systemic therapy. A: First-line. B: Second-line. C: Third-line. D: Fourth line.Fig. 2. Kaplan-Meier curves of overall survival and log-rank test results after start of primary treatment. A: Overall. B: Reason for systemic therapy. C: Reason for systemic therapy in more detail. D: Second-line therapy.Supplementary file2 (DOCX 278 KB)

## Data Availability

The data supporting the findings of this study are available from the corresponding author upon reasonable request.

## References

[CR1] Adelstein D, Gillison ML, Pfister DG, Spencer S, Adkins D, Brizel DM, Burtness B, Busse PM, Caudell JJ, Cmelak AJ, Colevas AD, Eisele DW, Fenton M, Foote RL, Gilbert J, Haddad RI, Hicks WL, Hitchcock YJ, Jimeno A, Leizman D, Lydiatt WM, Maghami E, Mell LK, Mittal BB, Pinto HA, Ridge JA, Rocco J, Rodriguez CP, Shah JP, Weber RS, Witek M, Worden F, Yom SS, Zhen W, Burns JL, Darlow SD (2017). NCCN guidelines insights: head and neck cancers, version 2.2017. J Natl Compr Canc Netw.

[CR2] Bauml J, Seiwert TY, Pfister DG, Worden F, Liu SV, Gilbert J, Saba NF, Weiss J, Wirth L, Sukari A, Kang H, Gibson MK, Massarelli E, Powell S, Meister A, Shu X, Cheng JD, Haddad R (2017). Pembrolizumab for platinum- and cetuximab-refractory head and neck cancer: results from a single-arm. Phase II Study J Clin Oncol.

[CR3] Bootz F (2020) S3-Leitlinie Diagnostik, Therapie und Nachsorge des Larynxkarzinoms. HNO. 10.1007/s00106-020-00908-y 10.1007/s00106-020-00908-y 32789706

[CR4] Burtness B, Harrington KJ, Greil R, Soulieres D, Tahara M, de Castro G, Psyrri A, Baste N, Neupane P, Bratland A, Fuereder T, Hughes BGM, Mesia R, Ngamphaiboon N, Rordorf T, Wan Ishak WZ, Hong RL, Gonzalez Mendoza R, Roy A, Zhang Y, Gumuscu B, Cheng JD, Jin F, Rischin D, Investigators K- (2019). Pembrolizumab alone or with chemotherapy versus cetuximab with chemotherapy for recurrent or metastatic squamous cell carcinoma of the head and neck (KEYNOTE-048): a randomised, open-label, phase 3 study. Lancet.

[CR5] Charlson M, Szatrowski TP, Peterson J, Gold J (1994). Validation of a combined comorbidity index. J Clin Epidemiol.

[CR6] Chow LQM (2020). Head and neck cancer. N Engl J Med.

[CR7] Cohen EEW, Soulieres D, Le Tourneau C, Dinis J, Licitra L, Ahn MJ, Soria A, Machiels JP, Mach N, Mehra R, Burtness B, Zhang P, Cheng J, Swaby RF, Harrington KJ, investigators K- (2019). Pembrolizumab versus methotrexate, docetaxel, or cetuximab for recurrent or metastatic head-and-neck squamous cell carcinoma (KEYNOTE-040): a randomised, open-label, phase 3 study. Lancet.

[CR8] David GP, Sharon S, David A, Douglas A, Yoshimi A, David MB, Justine YB, Paul MB, Jimmy JC, Anthony JC, Colevas AD, David WE, Moon F, Robert LF, Thomas G, Maura LG, Robert IH, Wesley LH, Ying JH, Antonio J, Debra L, Ellie M, Loren KM, Bharat BM, Harlan AP, John AR, James WR, Cristina PR, Jatin PS, Randal SW, Gregory W, Matthew W, Frank W, Sue SY, Weining Z, Jennifer LB, Susan DD (2020). Head and neck cancers, version 2.2020, NCCN clinical practice guidelines in oncology. J Natl Compr Canc Netw.

[CR9] Dittberner A, Friedl B, Wittig A, Buentzel J, Kaftan H, Boeger D, Mueller AH, Schultze-Mosgau S, Schlattmann P, Ernst T, Guntinas-Lichius O (2020). Gender disparities in epidemiology, treatment, and outcome for head and neck cancer in Germany: a population-based long-term analysis from 1996 to 2016 of the thuringian cancer registry. Cancers (Basel).

[CR10] El Rassy E, Assi T, Bakouny Z, El Karak F, Pavlidis N, Ghosn M (2019). Comparison of second-line treatments of recurrent and/or metastatic squamous cell carcinoma of the head and neck. Future Oncol.

[CR11] Ferris RL, Blumenschein G, Fayette J, Guigay J, Colevas AD, Licitra L, Harrington K, Kasper S, Vokes EE, Even C, Worden F, Saba NF, Iglesias Docampo LC, Haddad R, Rordorf T, Kiyota N, Tahara M, Monga M, Lynch M, Geese WJ, Kopit J, Shaw JW, Gillison ML (2016). Nivolumab for recurrent squamous-cell carcinoma of the head and neck. N Engl J Med.

[CR12] Ferris RL, Blumenschein G, Fayette J, Guigay J, Colevas AD, Licitra L, Harrington KJ, Kasper S, Vokes EE, Even C, Worden F, Saba NF, Docampo LCI, Haddad R, Rordorf T, Kiyota N, Tahara M, Lynch M, Jayaprakash V, Li L, Gillison ML (2018). Nivolumab vs investigator’s choice in recurrent or metastatic squamous cell carcinoma of the head and neck: 2-year long-term survival update of CheckMate 141 with analyses by tumor PD-L1 expression. Oral Oncol.

[CR13] Fisher MD, Fernandes AW, Olufade TO, Miller PJ, Walker MS, Fenton M (2018). Effectiveness outcomes in patients with recurrent or refractory head and neck cancers: retrospective analysis of data from a community oncology database. Clin Ther.

[CR14] Grunwald V, Chirovsky D, Cheung WY, Bertolini F, Ahn MJ, Yang MH, Castro G, Berrocal A, Sjoquist K, Kuyas H, Auclair V, Guillaume X, Joo S, Shah R, Harrington K, Glance H, Investigators NS (2020). Global treatment patterns and outcomes among patients with recurrent and/or metastatic head and neck squamous cell carcinoma: Results of the GLANCE H&N study. Oral Oncol.

[CR15] Guigay J, Tahara M, Licitra L, Keilholz U, Friesland S, Witzler P, Mesia R (2019). The evolving role of Taxanes in combination with cetuximab for the treatment of recurrent and/or metastatic squamous cell carcinoma of the head and neck: evidence, advantages, and future directions. Front Oncol.

[CR16] Guntinas-Lichius O, Wendt TG, Kornetzky N, Buentzel J, Esser D, Boeger D, Mueller A, Schultze-Mosgau S, Schlattmann P, Schmalenberg H (2014). Trends in epidemiology and treatment and outcome for head and neck cancer: a population-based long-term analysis from 1996 to 2011 of the Thuringian cancer registry. Oral Oncol.

[CR17] La EM, Smyth EN, Talbird SE, Li L, Kaye JA, Lin AB, Bowman L (2018). Treatment patterns and health care resource use in patients receiving multiple lines of therapy for metastatic squamous cell carcinoma of the head and neck in the United Kingdom. Eur J Cancer Care.

[CR18] Laban S, Kimmeyer J, Knecht R, Hoffmann TK, Busch CJ, Veit JA, Möckelmann N, Kurzweg T (2016). Palliative treatment standards for head and neck squamous cell carcinoma: survey of clinical routine in German-speaking countries. HNO.

[CR19] Misiukiewicz K, Camille N, Gupta V, Bakst R, Teng M, Miles B, Genden E, Sikora A, Posner M (2014). The role of HPV status in recurrent/metastatic squamous cell carcinoma of the head and neck. Clin Adv Hematol Oncol.

[CR20] NIH U.S. National Library of Medicine (2020) Study of Nivolumab in Combination With Ipilimumab Compared to the Standard of Care (Extreme Regimen) as First Line Treatment in Patients With Recurrent or Metastatic Squamous Cell Carcinoma of the Head and Neck (CheckMate 651) NCT02741570. Accessed September, 20th 2020

[CR21] NIH U.S. National Library of Medicine (2020) Phase III Open Label Study of MEDI 4736 With/Without Tremelimumab Versus Standard of Care (SOC) in Recurrent/Metastatic Head and Neck Cancer (KESTREL) NCT02551159. Accessed September 20th 2020

[CR22] Oosting SF, Haddad RI (2019). Best practice in systemic therapy for head and neck squamous cell carcinoma. Front Oncol.

[CR23] Pai SI, Faivre S, Licitra L, Machiels JP, Vermorken JB, Bruzzi P, Gruenwald V, Giglio RE, Leemans CR, Seiwert TY, Soulieres D (2019). Comparative analysis of the phase III clinical trials of anti-PD1 monotherapy in head and neck squamous cell carcinoma patients (CheckMate 141 and KEYNOTE 040). J Immunother Cancer.

[CR24] Schwam ZG, Burtness B, Yarbrough WG, Mehra S, Husain Z, Judson BL (2015). National treatment patterns in patients presenting with Stage IVC head and neck cancer: analysis of the National Cancer Database. Cancer Med.

[CR25] Vermorken JB, Mesia R, Rivera F, Remenar E, Kawecki A, Rottey S, Erfan J, Zabolotnyy D, Kienzer HR, Cupissol D, Peyrade F, Benasso M, Vynnychenko I, De Raucourt D, Bokemeyer C, Schueler A, Amellal N, Hitt R (2008). Platinum-based chemotherapy plus cetuximab in head and neck cancer. N Engl J Med.

[CR26] Wolff KD, Follmann M, Nast A (2012). The diagnosis and treatment of oral cavity cancer. Deutsches Arzteblatt Int.

