# Supplementary material for: Systemic therapy for recurrent and/or metastatic head and neck cancer: a population-based healthcare research study in Thuringia, Germany
Source: J Cancer Res Clin Oncol. 2021 Jan 31;147(9):2625–35. doi: 10.1007/s00432-021-03535-4 (PMC8310840; doi:10.1007/s00432-021-03535-4)
Supplement: Supplementary file 1 — Supplementary file1 (DOCX 77 KB) [file 432_2021_3535_MOESM1_ESM.docx]

**Supplement Tables**

**Supplement Table 1**

| **Supplement Table 1.** Characteristics of the primary tumor and the recurrent or metastatic tumor. | | |
| --- | --- | --- |
| **Parameter** | **N** | **%** |
| Timing of systemic therapy |  |  |
| Primary disease | 35 | 12.4 |
| Recurrent/progressive disease | 248 | 87.6 |
| Reason for systemic therapy |  |  |
| Locoregional recurrence | 112 | 39.6 |
| Progressive tumor | 69 | 24.4 |
| Distant metastasis recurrence | 67 | 23.7 |
| Primary distant metastatic tumor | 24 | 8.5 |
| Primary advanced locoregional tumor | 11 | 3.9 |
| Primary tumor site |  |  |
| Oropharynx | 121 | 42.8 |
| Hypopharynx | 61 | 21.6 |
| Oral cavity | 39 | 13.8 |
| Larynx | 30 | 10.6 |
| Nasopharynx | 9 | 3.2 |
| Salivary glands | 8 | 2.8 |
| Carcinoma of unknown primary | 6 | 2.1 |
| Nose/paranasal sinus | 6 | 2.1 |
| Ear | 3 | 1.1 |
| Histology |  |  |
| Squamous cell carcinoma | 241 | 85.2 |
| Other/not specified carcinoma | 42 | 14.8 |
| rUICC stage |  |  |
| I | 2 | 0.7 |
| II | 5 | 1.8 |
| III | 4 | 1.4 |
| IV | 272 | 96.1 |
| Prior primary curative therapy |  |  |
| Radiochemotherapy | 93 | 32.9 |
| Surgery and postoperative radiochemotherapy | 80 | 28.3 |
| Surgery | 35 | 12.4 |
| Surgery and postoperative radiotherapy | 31 | 11.0 |
| Radiotherapy | 8 | 2.8 |
| Induction chemotherapy and surgery | 1 | 0.4 |
| No curative therapy (primary systemic therapy) | 35 | 12.4 |
| Primary UICC stage |  |  |
| I | 17 | 6.0 |
| II | 12 | 4.2 |
| III | 29 | 10.2 |
| IV | 217 | 76.7 |
| Unknown | 8 | 2.8 |
|  | **Mean±SD** | **Median, range** |
| Interval primary treatment to 1^st^ line systemic therapy, months | 20.9±34.9 | 11, 0-250 |
| Interval primary to 1^st^ line systemic therapy, months  (without patients with primary systemic therapy) | 24.2±36.5 | 12, 1-250 |

SD = standard deviation

**Supplement Table 2**

| **Supplement table 2.** Distant metastasis sites. | | |
| --- | --- | --- |
| **Distant site** | **Primary distant metastatic M+**  **(N=24 patients)** | **Recurrent distant metastatic rM+**  **N=67 patients)** |
|  | N* | N* |
| PUL | 19 | 48 |
| OSS | 4 | 5 |
| HEP | 3 | 3 |
| ADR | 1 | - |
| PLE | 1 | - |
| SKI | 1 | 2 |
| PER | - | 1 |
| MAR | - | 1 |
| BRA | - | 1 |
| OTH | - | 8 |

*Number of distant metastasis was larger than number of patients because some patients had distant metastases at several sites. M1 categories: Pulmonary PUL; Osseous OSS; Hepatic HEP; Brain BRA; Bone marrow MAR; Pleura PLE; Peritoneum PER; Adrenal ADR; Distant skin SKI Other OTH.

**Supplement Table 3**

| **Supplement table 3.** Chemotherapy/antibody therapy as part of prior curative therapy (N=174 patients). | | |
| --- | --- | --- |
| **Drug** | **N** | **%*** |
| Platinum* | 149 | 52.7 |
| 5-fluorouracil | 106 | 37.5 |
| Docetaxel | 4 | 1.4 |
| Cetuximab | 22 | 7.8 |
| No prior chemotherapy (chemotherapy naïve) | 109 | 38.5 |

*sum was higher than 100% because some patients received several drugs. *Cisplatin or carboplatin

**Supplement Table 4**

| **Supplement Table 4.** Comparison of patients treated with the EXTREME protocol versus other protocol as first-line systemic therapy*. | | | |
| --- | --- | --- | --- |
| **Parameter** | **EXTREME**  **N=93** | **Not EXTREME**  **N=167** | **p** |
| **Baseline** |  |  |  |
| Gender |  |  | 0.213 |
| Male | 80 | 152 |  |
| Female | 13 | 15 |  |
| Age (median) |  |  | 0.702 |
| ≤60 years | 47 | 88 |  |
| >60 years | 46 | 78 |  |
| CCI |  |  | **0.048** |
| 0-1 | 88 | 145 |  |
| 2+ | 5 | 22 |  |
| Chronic kidney disease |  |  | 0.109 |
| Yes | 5 | 19 |  |
| No | 88 | 148 |  |
| Tobacco smoking |  |  | **<0.0001** |
| Yes | 54 | 62 |  |
| No | 32 | 99 |  |
| Alcohol drinking |  |  | **0.005** |
| Yes | 46 | 57 |  |
| No | 38 | 102 |  |
| Time of 1^st^ line systemic therapy |  |  | 0.565 |
| Primary disease | 11 | 24 |  |
| Recurrent/progressive disease | 82 | 143 |  |
| Reason for systemic therapy |  |  | 0.081 |
| Progress | 29 | 33 |  |
| Locoregional recurrence | 35 | 66 |  |
| Metastatic recurrence | 18 | 44 |  |
| Primary advanced locoregional tumor | 1 | 10 |  |
| Primarily metastatic | 10 | 14 |  |
| Progress |  |  | **0.038** |
| Yes | 29 | 33 |  |
| No | 64 | 134 |  |
| Locoregional recurrence |  |  | 0.765 |
| Yes | 36 | 66 |  |
| No | 58 | 101 |  |
| Metastatic recurrence |  |  | 0.205 |
| Yes | 18 | 44 |  |
| No | 75 | 123 |  |
| Primarily metastatic |  |  | 0.527 |
| Yes | 10 | 14 |  |
| No | 83 | 153 |  |
| Primary tumor site |  |  | 0.498 |
| Nasopharynx | 3 | 5 |  |
| Oropharynx | 36 | 76 |  |
| Hypopharynx | 25 | 31 |  |
| Larynx | 11 | 13 |  |
| Carcinoma of unknown primary | 1 | 5 |  |
| Salivary glands | 2 | 6 |  |
| Oral cavity | 12 | 25 |  |
| Nose/paranasal sinus | 1 | 5 |  |
| Ear | 2 | 1 |  |
| Oropharynx |  |  | 0.289 |
| Yes | 36 | 76 |  |
| No | 57 | 91 |  |
| Hypopharynx |  |  | 0.118 |
| Yes | 25 | 31 |  |
| No | 68 | 136 |  |
| Larynx |  |  | 0.280 |
| Yes | 11 | 13 |  |
| No | 82 | 154 |  |
| Oral cavity |  |  | 0.648 |
| Yes | 12 | 25 |  |
| No | 81 | 142 |  |
| rUICC stage |  |  | 0.061 |
| I | 1 | 1 |  |
| II | 0 | 5 |  |
| III | 0 | 3 |  |
| IV | 92 | 158 |  |
| Histology |  |  | 0.082 |
| Squamous cell carcinoma | 82 | 138 |  |
| Other/not specified carcinoma | 2 | 12 |  |
| **Primary treatment** |  |  |  |
| Surgery as part of primary treatment |  |  | 0.361 |
| Yes | 52 | 83 |  |
| No | 41 | 83 |  |
| Radiotherapy as part of primary treatment |  |  | 0.622 |
| Yes | 70 | 121 |  |
| No | 23 | 46 |  |
| Polychemotherapy as part of primary treatment | |  | **<0.0001** |
| Yes | 17 | 76 |  |
| No chemotherapy or single chemotherapy | 76 | 87 |  |
| Platinum** treatment of the primary tumor |  |  | 0.547 |
| Yes | 46 | 87 |  |
| No | 47 | 76 |  |
| 5-FU treatment of the primary tumor |  |  | **<0.0001** |
| Yes | 16 | 75 |  |
| No | 77 | 88 |  |
| Docetaxel treatment of the primary tumor |  |  | 0.272 |
| Yes | 2 | 1 |  |
| No | 91 | 162 |  |
| Cetuximab treatment of the primary tumor |  |  | 0.341 |
| Yes | 8 | 9 |  |
| No | 85 | 154 |  |
| Chemotherapy naïve |  |  | 0.704 |
| Yes | 39 | 66 |  |
| No | 54 | 101 |  |
| **1^st^ line systemic therapy** |  |  |  |
| Interval primary treatment to systemic therapy (median) | |  | 0.150 |
| < 11 months | 52 | 73 |  |
| ≥ 11 months | 41 | 84 |  |
| Additional palliative surgery to 1^st^ line |  |  | 0.073 |
| Yes | 3 | 16 |  |
| No | 83 | 146 |  |
| Additional palliative radiotherapy to 1^st^ line |  |  | **0.020** |
| Yes | 12 | 45 |  |
| No | 71 | 117 |  |

*N=260; regime for N=23 unknown; **Cisplatin or carboplatin; CCI = Charlson comorbidity index

**Supplement Table 5**

| **Supplement Table 5.** Independent factors associated with the selection of the EXTREME protocol as first-line systemic therapy (OR>1: pro EXTREME) | | | | |
| --- | --- | --- | --- | --- |
| **Parameter** | **OR** | **95% CI**  **lower** | **95% CI**  **upper** | **p** |
| **Baseline** |  |  |  |  |
| CCI |  |  |  |  |
| 2+ | Referent | 1 |  |  |
| 0-1 | 3.205 | 0.977 | 10.526 | 0.055 |
| Tobacco smoking |  |  |  |  |
| No | Referent | 1 |  |  |
| Yes | 2.189 | 0.954 | 5.022 | 0.064 |
| Alcohol drinking |  |  |  |  |
| No | Referent | 1 |  |  |
| Yes | 1.314 | 0.573 | 3.015 | 0.519 |
| Progress |  |  |  |  |
| No | Referent | 1 |  |  |
| Yes | 2.665 | 1.283 | 5.536 | **0.009** |
| **Primary treatment** |  |  |  |  |
| Polychemotherapy as part of primary treatment |  |  |  |  |
| No chemotherapy or single chemotherapy | Referent | 1 |  |  |
| Yes | 2.093 | 0.124 | 35.275 | 0.608 |
| 5-FU treatment of the primary tumor |  |  |  |  |
| Yes | Referent | 1 |  |  |
| No | 10.753 | 0.591 | 200.000 | 0.109 |
| **1^st^ line systemic therapy** |  |  |  |  |
| Additional palliative radiotherapy to 1^st^ line |  |  |  |  |
| Yes | Referent | 1 |  |  |
| No | 2.160 | 0.979 | 4.762 | 0.056 |

CCI = Charlson comorbidity index; OR = odds ratio; CI = confidence interval

**Supplement Table 6**

| **Supplement Table 6.** Reasons for termination of the systemic therapy. | | | | | | | | | | | |
| --- | --- | --- | --- | --- | --- | --- | --- | --- | --- | --- | --- |
|  | **1^st^ line** |  | **2^nd^ line** |  | **3^rd^ line** |  | **4^th^ line** |  | **5^th^ line** |  |  |
| **Parameter** | **N** | **%** | **N** | **%** | **N** | **%** | **N** | **%** | **N** | **%** |  |
| No systemic therapy |  |  | 191 | 67.5 | 246 | 86.9 | 269 | 95.1 | 280 | 98.9 |  |
| No termination, under therapy | 55 | 19.4 | 27 | 9.5 | 12 | 4.2 | 4 | 1.4 | 2 | 0.7 |  |
| Death under therapy | 30 | 10.6 | 8 | 2.8 | 8 | 2.8 | 2 | 0.7 | 1 | 0.4 |  |
| Progressive disease | 67 | 23.7 | 31 | 11.0 | 12 | 4.2 | 6 | 2.1 |  |  |  |
| Wish of the patient | 15 | 5.3 | 6 | 2.1 | 1 | 0.4 | 1 | 0.4 |  |  |  |
| Poor general health | 38 | 13.4 | 7 | 2.5 | 2 | 0.7 | 1 | 0.4 |  |  |  |
| Allergic reaction, drug intolerance | 18 | 6.4 | 3 | 1.1 |  |  |  |  |  |  |  |
| Other side effects | 8 | 2.8 | 4 | 1.4 | 1 | 0.4 |  |  |  |  |  |
| Planned termination, end of protocol | 20 | 7.1 | 6 | 2.1 | 1 | 0.4 |  |  |  |  |  |
| Other acute disease | 2 | 0.7 |  |  |  |  |  |  |  |  |  |
| Reason unknown | 29 | 10.2 |  |  |  |  |  |  |  |  |  |

**Supplement Table 7**

| **Supplement Table 7.** Data on lines, cycles and agents. | | |
| --- | --- | --- |
| **Parameter** | **Mean±SD** | **Median, range** |
| Sum of cycles of all received lines |  |  |
| Platinum* | 3.9±2.9 | 2, 0-20 |
| Cetuximab | 3.2±4.8 | 2, 0-41 |
| 5-FU | 1.8±2.0 | 1, 0-8 |
| Taxane | 1.0±2.1 | 0, 0-12 |
| Nivolumab | 1.0±3.3 | 0, 0-21 |
| Other agent | 0.2±0.8 | 0, 0-6 |
|  | **N** | **%** |
| Number of lines |  |  |
| Only first-line | 191 | 67.5 |
| First and second-line | 55 | 19.4 |
| First to third-line | 23 | 8.1 |
| First to fourth-line | 11 | 3.9 |
| First to fifth line | 3 | 1.1 |
| Patient received in any line |  |  |
| Platinum* | 210 | 80.8 |
| Cetuximab | 198 | 70.0 |
| 5-FU | 142 | 50.2 |
| Taxane | 75 | 26.5 |
| Nivolumab | 42 | 14.8 |
| Other agent | 19 | 6.7 |
| Unknown |  |  |
| Number of different agents applied |  |  |
| 1 | 44 | 15.5 |
| 2 | 60 | 21.2 |
| 3 | 114 | 40.3 |
| 4 | 33 | 11.7 |
| 5 | 6 | 2.1 |
| 6 | 3 | 1.1 |
| Unknown | 23 | 8.1 |

*Cisplatin or carboplatin

**Supplement Table 8**

| **Supplement Table 8.** Comparison of patients receiving a 2^nd^ line systemic therapy versus no 2^nd^ line systemic therapy.* | | | |
| --- | --- | --- | --- |
| **Parameter** | **2^nd^ line**  **N=92** | **No 2^nd^ line**  **N=77** | **p** |
| **Baseline** |  |  |  |
| Gender |  |  | 0.650 |
| Male | 83 | 71 |  |
| Female | 9 | 6 |  |
| Age (median) |  |  | 0.125 |
| ≤59 years | 51 | 34 |  |
| >59 years | 42 | 43 |  |
| CCI |  |  | 0.867 |
| 0-1 | 82 | 68 |  |
| 2+ | 10 | 9 |  |
| Chronic kidney disease |  |  | 0.548 |
| Yes | 11 | 7 |  |
| No | 81 | 70 |  |
| Tobacco smoking |  |  | **0.039** |
| Yes | 50 | 30 |  |
| No | 37 | 43 |  |
| Alcohol drinking |  |  | 0.641 |
| Yes | 42 | 33 |  |
| No | 45 | 41 |  |
| Timing of 1^st^ line systemic therapy |  |  | 0.865 |
| Primary disease | 14 | 11 |  |
| Recurrent/progressive disease | 78 | 66 |  |
| Reason for 1^st^ line systemic therapy |  |  | 0.910 |
| Progress | 19 | 17 |  |
| Locoregional recurrence | 35 | 31 |  |
| Metastatic recurrence | 24 | 18 |  |
| Primary advanced locoregional tumor | 4 | 5 |  |
| Primarily metastatic | 10 | 6 |  |
| Progress as reason for 1^st^ line |  |  | 0.822 |
| Yes | 19 | 17 |  |
| No | 73 | 60 |  |
| Locoregional recurrence as reason for 1^st^ line | |  | 0.769 |
| Yes | 35 | 31 |  |
| No | 57 | 46 |  |
| Metastatic recurrence as reason for 1^st^ line |  |  | 0.685 |
| Yes | 24 | 18 |  |
| No | 68 | 59 |  |
| Primarily metastatic as reason for 1^st^ line |  |  | 0.496 |
| Yes | 10 | 6 |  |
| No | 82 | 71 |  |
| Primary tumor site |  |  | 0.070 |
| Nasopharynx | 2 | 4 |  |
| Oropharynx | 46 | 28 |  |
| Hypopharynx | 19 | 16 |  |
| Larynx | 5 | 8 |  |
| Carcinoma of unknown primary | 1 | 3 |  |
| Salivary glands | 6 | 1 |  |
| Oral cavity | 8 | 15 |  |
| Nose/paranasal sinus | 2 | 2 |  |
| Ear | 3 | 0 |  |
| Oropharynx |  |  | 0.075 |
| Yes | 46 | 28 |  |
| No | 46 | 49 |  |
| Hypopharynx |  |  | 0.984 |
| Yes | 19 | 16 |  |
| No | 73 | 61 |  |
| Larynx |  |  | 0.229 |
| Yes | 5 | 8 |  |
| No | 87 | 69 |  |
| Oral cavity |  |  | **0.042** |
| Yes | 8 | 15 |  |
| No | 84 | 62 |  |
| rUICC stage |  |  | 0.236 |
| I | 0 | 2 |  |
| II | 1 | 2 |  |
| III | 0 | 1 |  |
| IV | 91 | 72 |  |
| Histology |  |  | 0.637 |
| Squamous cell carcinoma | 80 | 65 |  |
| Other/not specified carcinoma | 12 | 12 |  |
| **Primary treatment** |  |  |  |
| Surgery as part of primary treatment |  |  |  |
| Yes | 48 | 42 | 0.758 |
| No | 44 | 35 |  |
| Radiotherapy as part of primary treatment |  |  | 0.383 |
| Yes | 70 | 54 |  |
| No | 22 | 23 |  |
| Polychemotherapy as part of primary treatment | |  | 0.988 |
| Yes | 35 | 29 |  |
| No chemotherapy or single chemotherapy | 57 | 47 |  |
| Platinum** treatment of the primary tumor |  |  | 0.631 |
| Yes | 45 | 40 |  |
| No | 47 | 36 |  |
| 5-FU treatment of the primary tumor |  |  | 0.988 |
| Yes | 35 | 29 |  |
| No | 57 | 48 |  |
| Docetaxel treatment of the primary tumor |  |  | NA |
| Yes | 0 | 0 |  |
| No | 92 | 77 |  |
| Cetuximab treatment of the primary tumor |  |  | 0.796 |
| Yes | 7 | 5 |  |
| No | 85 | 71 |  |
| Chemotherapy naïve |  |  | 0.966 |
| Yes | 37 | 31 |  |
| No | 55 | 46 |  |
| **1^st^ line systemic therapy** |  |  |  |
| Interval primary treatment to 1^st^ line therapy (median) | |  | 0.933 |
| < 10 months | 45 | 38 |  |
| ≥ 10 months | 47 | 39 |  |
| Additional palliative surgery to 1^st^ line |  |  | 0.546 |
| Yes | 6 | 7 |  |
| No | 85 | 70 |  |
| Additional palliative radiotherapy to 1^st^ line |  |  | 0.778 |
| Yes | 21 | 19 |  |
| No | 72 | 58 |  |
| Reasons for termination of 1^st^ line therapy |  |  | **<0.0001** |
| Progressive disease | 42 | 25 |  |
| Wish of the patient | 1 | 14 |  |
| Poor general health | 12 | 26 |  |
| Allergic reaction, drug intolerance | 14 | 4 |  |
| Other side effects | 6 | 2 |  |
| Planned termination, end of protocol | 14 | 6 |  |
| Other acute disease | 2 | 0 |  |
| Reasons for termination of 1^st^ line therapy |  |  | 0.143 |
| Progressive disease | 40 | 25 |  |
| Other reason, not progressive disease | 52 | 52 |  |
| Reasons for termination of 1^st^ line therapy |  |  | **0.001** |
| Poor general health | 12 | 26 |  |
| Other reason, not poor general health | 80 | 51 |  |
| Duration of 1^st^ line systemic therapy (median)* | |  | 0.943 |
| ≤2.8 months | 45 | 38 |  |
| >2.8 months | 44 | 38 |  |
| Number of agents of 1^st^ line therapy |  |  | **0.022** |
| Single agent regime | 10 | 21 |  |
| Two agent regime | 37 | 27 |  |
| Three agent regime | 45 | 29 |  |
| Platinum** as part of 1^st^ line regime |  |  | **0.013** |
| Yes | 78 | 53 |  |
| No | 14 | 24 |  |
| Cetuximab as part of 1^st^ line regime |  |  | 0.497 |
| Yes | 65 | 58 |  |
| No | 27 | 19 |  |
| 5-FU as part of 1^st^ line regime |  |  | 0.312 |
| Yes | 49 | 35 |  |
| No | 43 | 42 |  |
| Taxane as part of 1^st^ line regime |  |  | 0.179 |
| Yes | 22 | 12 |  |
| No | 70 | 65 |  |
| Nivolumab as part of 1^st^ line regime |  |  | 0.232 |
| Yes | 1 | 3 |  |
| No | 91 | 74 |  |
| EXTREME regime as 1^st^ line therapy |  |  | 0.666 |
| Yes | 34 | 26 |  |
| No | 58 | 51 |  |

*only patients with completed 1^st^ line systemic therapy were included, patients still under 1^st^ line therapy or death under 1^st^ line therapy were excluded; **cisplatin or carboplatin; CCI = Charlson comorbidity index

**Supplement Table 9**

| **Supplement Table 9.** Independent factors associated with decision for a 2^nd^ line systemic therapy* (OR>1: pro 2^nd^ line) | | | | | |
| --- | --- | --- | --- | --- | --- |
| **Parameter** | **OR** | **95% CI***  **lower** | **95% CI**  **upper** | **p** |  |
| Tobacco smoking |  |  |  |  |  |
| No | Referent | 1 |  |  |  |
| Yes | 1.738 | 0.871 | 3.467 | 0.117 |  |
| Oral cavity |  |  |  |  |  |
| No | Referent | 1 |  |  |  |
| Yes | 1.912 | 0.948 | 3.854 | 0.070 |  |
| Reasons for termination of 1^st^ line therapy |  |  |  |  |  |
| Poor general health | Referent | 1 |  |  |  |
| Other reason, not poor general health | 1.912 | 0.948 | 3.854 | **0.001** |  |
| Number of agents of 1^st^ line therapy | 1.208 | 0.665 | 2.194 | 0.535 |  |
| Platinum** as part of 1^st^ line regime |  |  |  |  |  |
| No | Referent | 1 |  |  |  |
| Yes | 2.863 | 1.009 | 8.126 | **0.048** |  |

*only patients with completed 1^st^ line systemic therapy were included, patients still under 1^st^ line therapy or death under 1^st^ line therapy were excluded; OR = odds ratio; CI = confidence interval

**Supplement Table 10**

| **Supplement Table 10.** Follow up data and live status | | |
| --- | --- | --- |
| **Parameter** | **N** | **%** |
| Patients alive | 192 | 67.8 |
| Patients dead | 91 | 32.2 |
|  | **Mean±SD** | **Median, range** |
| Follow-up in months since primary diagnosis, months | 29.4±35.1 | 18.2, 1-163 |
| Follow-up in months since primary diagnosis of patients alive, months | 31.5±38.7 | 19.6, 1-163 |
| Follow-up in months since start of 1^st^ line systemic therapy, months | 8.6±9.7 | 5.5, 0-68 |
| Follow-up in months since start of 2^nd^ line systemic therapy, months | 8.2±9.1 | 6.0, 0-60 |
| Follow-up in months since start of 3^rd^ line systemic therapy, months | 6.2±6.1 | 4, 0-25 |
| Follow-up in months since start of 4^th^ line systemic therapy, months | 5.3±4.2 | 4.5, 0-13 |
| Follow-up in months since start of 5^th^ line systemic therapy, months | 2.0±2.0 | 2, 0-4 |
|  | **Median** | **95% CI** |
| Overall survival after initial diagnostic of primary tumor, months | 66.7 | 42.5-90.9 |
| Overall survival after start of 1^st^ line systemic therapy, months | 16.8 | 11.1-22.6 |
| Overall survival after start of 2^nd^ line systemic therapy, months* | 20.0 | 10.2-29.8 |
| Overall survival after start of 3^rd^ line systemic therapy, months* | 16.0 | 2.3-29.7 |
| Overall survival after start of 4^th^ line systemic therapy, months* | NA |  |
| Overall survival after start of 5^th^ line systemic therapy, months* | NA |  |

*of the patients receiving this line of systemic therapy; NA = not applicable

**Supplement Table 11**

| **Supplement Table 11.** Univariate analysis of prognostic factors for overall survival after start of 1^st^ line systemic therapy. | | | |
| --- | --- | --- | --- |
| **Parameter** | **6-month**  **overall survival rate** | **12-month**  **overall survival rate** | **p** |
| All | 73.3 | 59.6 |  |
| **Baseline** | | | |
| Gender |  |  | 0.475 |
| Male | 73.8 | 58.5 |  |
| Female | 69.4 | 69.4 |  |
| Age |  |  | 0.831 |
| ≤60 years | 73.0 | 59.8 |  |
| >60 years | 73.5 | 59.4 |  |
| CCI |  |  | 0.962 |
| 0-1 | 73.1 | 58.8 |  |
| 2+ | 73.5 | 66.9 |  |
| Tobacco smoking |  |  | 0.192 |
| Yes | 74.3 | 51.6 |  |
| No | 72.0 | 65.1 |  |
| Alcohol drinking |  |  | **0.002** |
| Yes | 67.1 | 46.3 |  |
| No | 78.0 | 68.6 |  |
| Oropharynx |  |  | 0.125 |
| Yes | 71.6 | 55.6 |  |
| No | 74.5 | 63.0 |  |
| Hypopharynx |  |  | 0.695 |
| Yes | 74.8 | 64.2 |  |
| No | 72.9 | 58.0 |  |
| Larynx |  |  | 0.392 |
| Yes | 68.7 | 53.4 |  |
| No | 73.8 | 60.5 |  |
| Oral cavity |  |  | 0.580 |
| Yes | 73.4 | 67.3 |  |
| No | 73.1 | 58.7 |  |
| **Primary treatment** |  |  |  |
| Surgery as part of primary treatment | | | 0.789 |
| Yes | 70.7 | 56.1 |  |
| No | 75.8 | 62.9 |  |
| Radiotherapy as part of primary treatment | |  | 0.813 |
| Yes | 71.0 | 53.3 |  |
| No | 74.1 | 62.0 |  |
| Polychemotherapy as part of primary treatment | |  | **0.013** |
| Yes | 82.7 | 73.4 |  |
| No chemotherapy or single chemotherapy | 67.1 | 50.8 |  |
| Platinum** treatment of the primary tumor | |  | 0.386 |
| Yes | 75.6 | 66.1 |  |
| No | 70.2 | 53.0 |  |
| 5-FU treatment of the primary tumor | |  | **0.008** |
| Yes | 83.7 | 74.3 |  |
| No | 66.7 | 50.5 |  |
| Taxane treatment of the primary tumor | |  | 0.001 |
| Yes | 33.3 | 33.3 |  |
| No | 73.6 | 59.9 |  |
| Cetuximab treatment of the primary tumor | |  | 0.888 |
| Yes | 73.1 | 59.4 |  |
| No | 72.2 | 60.2 |  |
| Chemotherapy naïve before1^st^ line systemic therapy | | | 0.180 |
| Yes | 68.2 | 49.6 |  |
| No | 76.5 | 66.9 |  |
| **1^st^ line systemic therapy** | | | |
| Timing of 1^st^ line systemic therapy | |  | 0.385 |
| Primary treatment | 72.4 | 48.8 |  |
| Recurrent/progressive disease | 73.5 | 61.9 |  |
| Progress |  |  | 0.802 |
| Yes | 73.2 | 63.3 |  |
| No | 73.3 | 58.4 |  |
| Locoregional recurrence | |  | 0.832 |
| Yes | 70.6 | 62.5 |  |
| No | 75.0 | 58.1 |  |
| Metastatic recurrence |  |  | 0.233 |
| Yes | 78.1 | 59.5 |  |
| No | 71.6 | 59.7 |  |
| Primarily metastatic |  |  | 0.895 |
| Yes | 72.1 | 56.8 |  |
| No | 73.4 | 60.0 |  |
| rUICC stage |  |  | 0.997 |
| I-III | 64.3 | 64.3 |  |
| IV | 73.5 | 59.6 |  |
| Interval primary treatment to 1^st^ systemic therapy (median) | | |  |
| < 11 months | 73.6 | 55.8 | 0.538 |
| ≥ 11 months | 74.4 | 65.7 |  |
| Number of agents of 1^st^ line regime | |  | **<0.0001** |
| Single agent regime | 84.5 | 75.6 |  |
| Two agent regime | 84.2 | 70.7 |  |
| Three agent regime | 59.9 | 44.4 |  |
| Platinum as part of 1^st^ line regime | |  | **0.015** |
| Yes | 70.6 | 54.9 |  |
| No | 82.8 | 78.2 |  |
| Cetuximab as part of 1^st^ line regime | |  | **0.047** |
| Yes | 69.5 | 56.1 |  |
| No | 82.7 | 67.9 |  |
| 5-FU as part of 1^st^ line regime | |  | **<0.0001** |
| Yes | 63.9 | 49.0 |  |
| No | 82.6 | 69.9 |  |
| Taxane as part of 1^st^ line regime | |  | 0.546 |
| Yes | 73.0 | 58.0 |  |
| No | 73.2 | 60.1 |  |
| Nivolumab as part of 1^st^ line regime | |  | 0.730 |
| Yes | 76.2 | 76.2 |  |
| No | 73.1 | 59.1 |  |
| EXTREME regime as 1^st^ line regime | |  | **<0.0001** |
| Yes | 59.5 | 42.2 |  |
| No | 80.9 | 69.3 |  |
| Palliative radiotherapy as part of 1^st^ line | |  | 0.165 |
| Yes | 84.8 | 70.8 |  |
| No | 74.2 | 58.4 |  |
| Palliative surgery as part of 1^st^ line | |  | 0.260 |
| Yes | 81.9 | 60.6 |  |
| No | 74.6 | 61.2 |  |
| Number of cycles in 1st line therapy | |  | **0.005** |
| ≤2 | 57.2 | 47.9 |  |
| >2 | 78.7 | 62.8 |  |
| Duration of 1^st^ line |  |  | **0.039** |
| <2.8 months | 74.2 | 60.7 |  |
| ≥2.8 months | 86.5 | 70.3 |  |
| Reasons for termination of 1^st^ line therapy | |  |  |
| Progressive disease | 87.6 | 69.0 | **0.006** |
| Other reason, not progressive disease | 67.8 | 56.7 |  |
| Reasons for termination of 1^st^ line therapy | |  | 0.814 |
| Poor general health | 73.6 | 69.3 |  |
| Other reason, not poor general health | 73.6 | 58.3 |  |
| **2^nd^ line and subsequent systemic therapies** | |  |  |
| 2^nd^ line systemic therapy | |  | **<0.0001** |
| Yes | 91.4 | 74.8 |  |
| No | 59.5 | 48.3 |  |
| Number of different lines of systemic therapy | |  | **0.003** |
| 1 | 59.9 | 49.1 |  |
| 2 | 91.1 | 61.1 |  |
| 3 | 90.7 | 90.7 |  |
| 4 | 90.9 | 80.8 |  |
| 5 | 100 | 100 |  |

CCI = Charlson comorbidity index

**Supplement Table 12**

| **Supplement Table 12.** Multivariable Cox regression of prognostic factors for overall survival after start of 1^st^ line systemic therapy (HR>1: worse OS) – model 1. | | | | | | | |
| --- | --- | --- | --- | --- | --- | --- | --- |
| **Parameter** |  | **HR** | **Lower 95%CI** | **Upper 95%CI** | **p** |  |  |
| **Baseline** | | | | | | | |
| Alcohol drinking | No | 1 | Reference | | | |  |
|  | Yes | 3.165 | 1.420 | 7.042 | **0.005** |  |  |
| **Primary treatment** |  |  |  | | | |  |
| Polychemotherapy as part of primary treatment | Yes | 1 | Reference | | | |  |
|  | No | 1.249 | 0.503 | 3.104 | 0.632 |  |  |
| **1^st^ line regime** | | | | | | | |
| Number of agents of 1^st^ line regime | 1 | 1 | Reference | | 0.867 |  |  |
|  | 2 | 1.343 | 0.338 | 5.328 | 0.675 |  |  |
|  | 3 | 1.743 | 0.225 | 13.530 | 0.595 |  |  |
| Platinum as part of 1^st^ line regime | Yes | 1 | Reference | | | |  |
|  | No | 2.556 | 0.534 | 12.233 | 0.240 |  |  |
| 5-FU as part of 1^st^ line regime | No | 1 | Reference | | | |  |
|  | Yes | 2.132 | 0.814 | 5.587 | 0.123 |  |  |
| Cetuximab as part of 1^st^ line regime | No | 1 | Reference | | | |  |
|  | Yes | 1.152 | 0.324 | 4.098 | 0.827 |  |  |
| Number of cycles in 1st line therapy (median) | >2 | 1 | Reference | | | |  |
|  | ≤2 | 1.229 | 0.523 | 2.885 | 0.636 |  |  |
| Duration of 1^st^ line (months; median) | ≥2.8 | 1 | Reference | | | |  |
|  | <2.8 | 1.439 | 0.594 | 3.487 | 0.420 |  |  |
| Reasons for termination of 1^st^ line therapy, | Yes | 1 | Reference | | | |  |
| progressive disease | No | 1.090 | 0.495 | 2.401 | 0.830 |  |  |
| **2^nd^ line and subsequent systemic therapies** | | | | | | | |
| 2^nd^ line systemic therapy | Yes | 1 | Reference | | | |  |
|  | No | 11.493 | 1.150 | 114.89 | **0.038** |  |  |
| Number of different lines of systemic therapy | 1 | 1 | Reference | | 0.579 |  |  |
|  | 2 | 0.208 | 0.021 | 2.103 | 0.184 |  |  |
|  | 3 | 0.298 | 0.028 | 3.130 | 0.313 |  |  |
|  | 4 | 0.201 | 0.013 | 3.171 | 0.254 |  |  |
|  | 5 | NA |  |  |  |  |  |

HR = hazard ratio; CI = confidence interval; PD = progressive disease; NA = not applicable

**Supplement Table 13**

| **Supplement Table 13.** Multivariable Cox regression of prognostic factors for overall survival after start of 1^st^ line systemic therapy (HR>1: worse OS) – model 2. | | | | | | |
| --- | --- | --- | --- | --- | --- | --- |
| **Parameter** |  | **HR** | **Lower 95%CI** | **Upper 95%CI** | **p** |  |
| **Baseline** | | | | | | |
| Alcohol drinking | No | 1 | Reference | | | |
|  | Yes | 2.375 | 1.471 | 3.831 | **<0.001** |  |
| **Primary treatment** |  |  |  | | | |
| Polychemotherapy as part of primary treatment | Yes | 1 | Reference | | | |
|  | No | 1.284 | 0.748 | 2.203 | 0.365 |  |
| **1st line regime** | | | | | | |
| Number of agents of 1^st^ line regime | 1 |  |  |  | 0.001 |  |
|  | 2 | 1.185 | 0.543 | 2.588 | 0.670 |  |
|  | 3 | 2.798 | 1.374 | 5.697 | **0.005** |  |
| **2^nd^ line and subsequent systemic therapies** | | | | | | |
| 2^nd^ line systemic therapy | Yes | 1 | Reference | | | |
|  | No | 3.425 | 2.082 | 5.635 | **<0.001** |  |

HR = hazard ratio; CI = confidence interval;

**Supplement Table 14**

| **Supplement Table 14.** Univariate analysis of prognostic factors for overall survival after start of 2^nd^ line systemic therapy.* | | | |
| --- | --- | --- | --- |
| **Parameter** | **6-month**  **overall survival rate** | **12-month**  **overall survival rate** | **p** |
| All | 75.0 | 59.5 |  |
| **Baseline** | | | |
| Gender |  |  | 0.747 |
| Male | 74.0 | 59.8 |  |
| Female | 83.3 | 62.5 |  |
| Age |  |  | 0.808 |
| ≤60 years | 72.9 | 60.3 |  |
| >60 years | 77.6 | 59.2 |  |
| CCI |  |  | 0.574 |
| 0-1 | 76.4 | 59.7 |  |
| 2+ | 63.5 | 63.5 |  |
| Tobacco smoking |  |  | **0.040** |
| Yes | 68.8 | 45.7 |  |
| No | 85.6 | 73.8 |  |
| Alcohol drinking |  |  | **0.004** |
| Yes | 61.0 | 39.4 |  |
| No | 91.5 | 76.7 |  |
| Oropharynx |  |  | 0.064 |
| Yes | 72.8 | 42.8 |  |
| No | 76.4 | 70.9 |  |
| Hypopharynx |  |  | 0.131 |
| Yes | 86.6 | 86.6 |  |
| No | 71.7 | 51.3 |  |
| Larynx |  |  | 0.738 |
| Yes | 80.0 | 80.0 |  |
| No | 74.3 | 57.1 |  |
| Oral cavity |  |  | 0.500 |
| Yes | 0.80 | 0.80 |  |
| No | 74.2 | 57.0 |  |
| **Primary treatment** |  |  |  |
| Surgery as part of primary treatment | | | 0.855 |
| Yes | 71.8 | 56.5 |  |
| No | 79.0 | 65.2 |  |
| Radiotherapy as part of primary treatment | |  | 0.860 |
| Yes | 78.9 | 61.8 |  |
| No | 76.4 | 52.4 |  |
| Polychemotherapy as part of primary treatment | |  | 0.475 |
| Yes | 79.0 | 71.1 |  |
| No chemotherapy or single chemotherapy | 72.3 | 54.1 |  |
| Platinum** treatment of the primary tumor | |  | 0.695 |
| Yes | 74.3 | 66.9 |  |
| No | 75.9 | 54.1 |  |
| 5-FU treatment of the primary tumor | |  | 0.475 |
| Yes | 79.0 | 71.1 |  |
| No | 72.3 | 54.1 |  |
| Taxane treatment of the primary tumor | |  | NA |
| Yes | NA | NA |  |
| No | 75.0 | 59.5 |  |
| Cetuximab treatment of the primary tumor | |  | 0.951 |
| Yes | 80.0 | 53.3 |  |
| No | 74.3 | 57.1 |  |
| Chemotherapy naïve after primary therapy | | | 0.705 |
| Yes | 72.4 | 56.3 |  |
| No | 76.5 | 67.1 |  |
| **1^st^ line systemic therapy** | | | |
| Timing of 1^st^ line systemic therapy | |  | 0.313 |
| Primary treatment | 74.0 | 49.4 |  |
| Recurrent/progressive disease | 79.2 | 67.7 |  |
| Progress |  |  | 0.920 |
| Yes | 80.4 | 62.5 |  |
| No | 77.9 | 67.6 |  |
| Locoregional recurrence | |  | 0.848 |
| Yes | 85.0 | 75.3 |  |
| No | 74.7 | 61.5 |  |
| Metastatic recurrence |  |  | 0.686 |
| Yes | 70.3 | 63.9 |  |
| No | 81.4 | 65.5 |  |
| Primarily metastatic |  |  | 0.300 |
| Yes | 64.8 | 64.8 |  |
| No | 80.2 | 66.3 |  |
| rUICC stage |  |  | NA |
| I-III | NA | NA |  |
| IV | 78.9 | 59.5 |  |
| Interval primary treatment to 1^st^ systemic therapy (median) | | | 0.161 |
| < 11 months | 78.8 | 63.8 |  |
| ≥ 11 months | 77.3 | 65.0 |  |
| Number of agents at 1^st^ line systemic therapy | |  | **0.026** |
| Single agent regime | 88.9 | 66.7 |  |
| Two agent regime | 79.7 | 74.0 |  |
| Three agent regime | 74.7 | 56.5 |  |
| Number of agents at 1^st^ line systemic therapy | |  | **0.007** |
| 1-2 agents | 82.1 | 73.0 |  |
| 3 agents | 74.7 | 56.5 |  |
| Platinum as part of 1^st^ line regime | |  | 0.156 |
| Yes | 75.9 | 63.1 |  |
| No | 91.7 | 78.6 |  |
| Cetuximab as part of 1^st^ line regime | |  | 0.476 |
| Yes | 78.8 | 67.1 |  |
| No | 77.9 | 62.3 |  |
| 5-FU as part of 1^st^ line regime | |  | 0.115 |
| Yes | 79.7 | 74.4 |  |
| No | 77.6 | 45.8 |  |
| Taxane as part of 1^st^ line regime | |  | 0.962 |
| Yes | 74.6 | 65.3 |  |
| No | 79.5 | 66.0 |  |
| Nivolumab as part of 1^st^ line regime | |  | NA |
| Yes | NA | NA |  |
| No | 78.2 | 62.2 |  |
| EXTREME regime as 1^st^ line regime | |  | **0.028** |
| Yes | 74.6 | 41.1 |  |
| No | 80.9 | 58.4 |  |
| Palliative radiotherapy as part of 1^st^ line | |  | 0.366 |
| Yes | 92.3 | 65.9 |  |
| No | 74.8 | 56.8 |  |
| Palliative surgery as part of 1^st^ line | |  | 0.217 |
| Yes | 44.4 | 44.4 |  |
| No | 80.8 | 66.5 |  |
| Number of cycles in 1st line therapy | |  | 0.377 |
| ≤2 | 79.0 | 72.9 |  |
| >2 | 75.8 | 53.9 |  |
| Duration of 1^st^ line |  |  | 0.413 |
| <2.8 months | 79.8 | 64.2 |  |
| ≥2.8 months | 75.2 | 50.3 |  |
| Reasons for termination of 1^st^ line therapy | |  | 0.949 |
| Progressive disease | 76.8 | 61.0 |  |
| Other reason, not progressive disease | 77.1 | 69.8 |  |
| Reasons for termination of 1^st^ line therapy | |  | 0.960 |
| Poor general health | 66.7 | 66.7 |  |
| Other reason, not poor general health | 79.9 | 65.7 |  |
| **2^nd^ line systemic therapy** | |  |  |
| Interval primary treatment to 2^nd^ line therapy (median) | | | 0.521 |
| ≤14 months | 76.2 | 56.2 |  |
| >14 months | 73.4 | 61.3 |  |
| Number of agents at 2^nd^ line systemic therapy | |  | **0.018** |
| Single agent regime | 75.7 | 59.9 |  |
| Two agent regime | 93.0 | 84.3 |  |
| Three agent regime | 50.5 | 18.9 |  |
| Platinum as part of 2^nd^ line regime | |  | 0.493 |
| Yes | 72.3 | 55.3 |  |
| No | 78.1 | 63.4 |  |
| Cetuximab as part of 2^nd^ line regime | |  | 0.694 |
| Yes | 77.9 | 69.8 |  |
| No | 71.4 | 60.8 |  |
| 5-FU as part of 2^nd^ line regime | |  | 0.163 |
| Yes | 63.9 | 53.3 |  |
| No | 79.3 | 62.3 |  |
| Taxane as part of 2^nd^ line regime | |  | 0.254 |
| Yes | 82.5 | 66.9 |  |
| No | 72.0 | 56.6 |  |
| Nivolumab as part of 2^nd^ line regime | |  | 0.353 |
| Yes | 59.3 | 59.3 |  |
| No | 77.4 | 60.8 |  |
| Palliative radiotherapy as part of 2^nd^ line | |  | 0.964 |
| Yes | 75.0 | 62.5 |  |
| No | 77.1 | 59.6 |  |
| Palliative surgery as part of 2^nd^ line | |  | 0.667 |
| Yes | 80.0 | 53.3 |  |
| No | 74.2 | 60.4 |  |
| Number of cycles in 2^nd^ line therapy | |  | 0.086 |
| ≤4 | 54.1 | 47.4 |  |
| >4 | 84.6 | 53.5 |  |
| Duration of 2^nd^ line |  |  | 0.141 |
| ≤3 months | 72.7 | 58.1 |  |
| >3 months | 89.5 | 63.7 |  |
| Reasons for termination of 2^nd^ line therapy | |  | 0.583 |
| Progressive disease | 70.4 | 62.5 |  |
| Other reason, not progressive disease | 76.6 | 36.1 |  |
| Reasons for termination of 2^nd^ line therapy | |  | **0.003** |
| Poor general health | 0 | 0 |  |
| Other reason, not poor general health | 73.8 | 56.1 |  |
| **3^rd^ line and subsequent systemic therapies** | |  |  |
| 3^rd^ line systemic therapy | |  | 0.064 |
| Yes | 91.7 | 71.1 |  |
| No | 59.6 | 49.8 |  |
| Number of different lines of systemic therapy | |  | 0.261 |
| 2 | 56.9 | 49.8 |  |
| 3 | 90.9 | 59.8 |  |
| 4 | 90.9 | 80.8 |  |
| 5 | 100 | 100 |  |

*Only patients undergoing 2^nd^ line therapy included; NA=not applicable; CCI=Charlson comorbidity index

**Supplement Table 15**

| **Supplement Table 15.** Multivariable Cox regression of prognostic factors for overall survival (OS) after start of 2^nd^ line systemic therapy (HR>1: worse OS).* | | | | | |
| --- | --- | --- | --- | --- | --- |
| **Parameter** |  | **HR** | **Lower 95%CI** | **Upper 95%CI** | **p** |
| **Baseline** |  |  |  | | |
| Tobacco smoking | No | 1 | Reference | | |
|  | Yes | 1.124 | 0.373 | 3.390 | 0.836 |
| Alcohol drinking | No | 1 | Reference | | |
|  | Yes | 2.331 | 0.787 | 6.897 | 0.127 |
| **1^st^ line regime** |  |  |  | | |
| Number of agents at 1^st^ line systemic therapy | 1 | 1 | Reference | | 0.846 |
|  | 2 | 0.999 | 0.197 | 5.070 | 0.999 |
|  | 3 | 1.446 | 0.235 | 8.909 | 0.691 |
| EXTREME regime as 1^st^ line regime | No | 1 | Reference | |  |
|  | Yes | 1.449 | 0.424 | 4.950 | 0.554 |
| **2^nd^ line regime** |  |  |  |  |  |
| Number of agents at 2^nd^ line systemic therapy | 1 | 1 | Reference | | 0.497 |
|  | 2 | 0.783 | 0.290 | 2.111 | 0.629 |
|  | 3 | 1.640 | 0.558 | | |
| Reasons for termination of 2^nd^ line therapy,  deterioration of general health | No | 1 | Reference | |  |
|  | Yes | 4.202 | 1.091 | 16.129 | **0.037** |

*Only patients undergoing 2^nd^ line therapy included
