# Supplementary figures and images for: Systemic therapy for recurrent and/or metastatic head and neck cancer: a population-based healthcare research study in Thuringia, Germany
Source: J Cancer Res Clin Oncol. 2021 Jan 31;147(9):2625–35. doi: 10.1007/s00432-021-03535-4 (PMC8310840; doi:10.1007/s00432-021-03535-4)

**Supplement Figures**

**Supplement Figure 1**


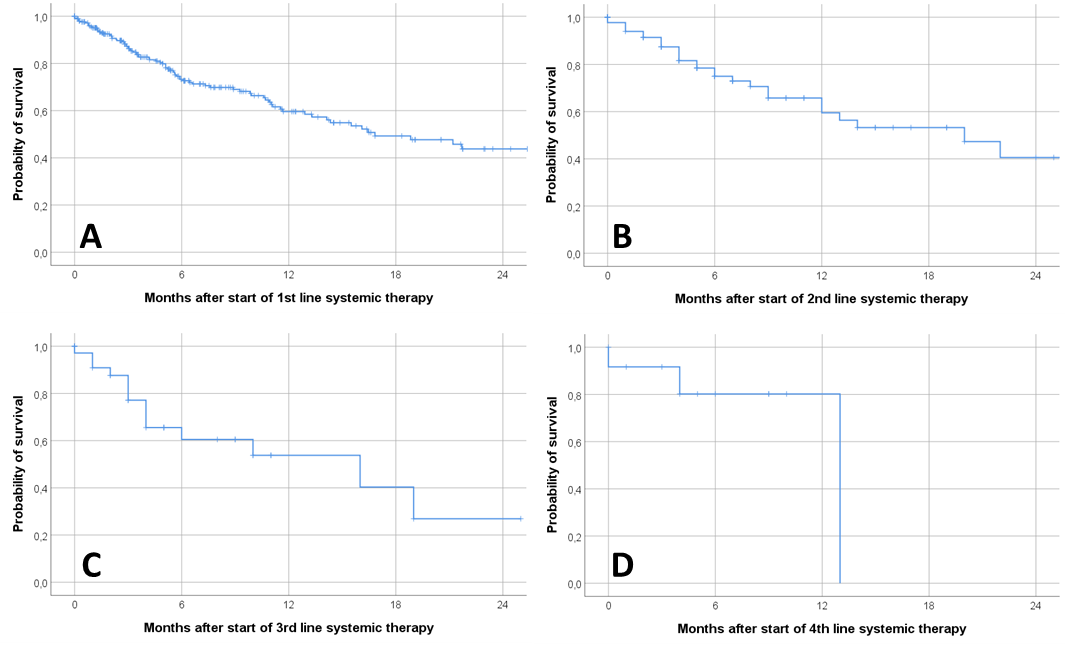


**Supplement Figure 2**


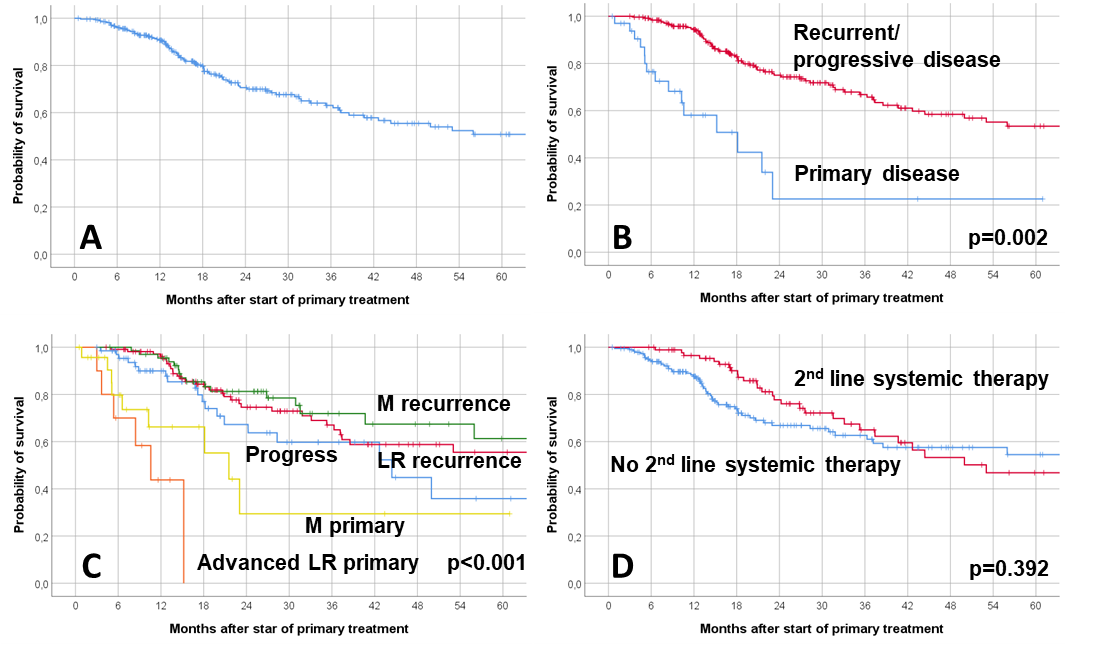

Supplement: Supplementary file 2 — Fig. 1. Kaplan-Meier curves of overall survival after start of different lines of systemic therapy. A: First-line. B: Second-line. C: Third-line. D: Fourth line.Fig. 2. Kaplan-Meier curves of overall survival and log-rank test results after start of primary treatment. A: Overall. B: Reason for systemic therapy. C: Reason for systemic therapy in more detail. D: Second-line therapy.Supplementary file2 (DOCX 278 KB) [file 432_2021_3535_MOESM2_ESM.docx]
